# Positive correlation between *Bax* and *Bcl-2* gene polymorphisms with the risk of endometriosis: A case-control study

**DOI:** 10.18502/ijrm.v22i6.16796

**Published:** 2024-08-05

**Authors:** Arefe Edalatian Kharrazi, Forough Forghani, Danial Jahantigh, Saeedeh Ghazaey Zidanloo, Mahnaz Rezaei, Mohsen Taheri

**Affiliations:** ^1^Department of Obstetrics and Gynecology, Zahedan University of Medical Sciences, Zahedan, Iran.; ^2^Department of Biology, Faculty of Sciences, University of Sistan and Baluchestan, Zahedan, Iran.; ^3^Genetics of Non-Communicable Disease Research Center, Zahedan University of Medical Sciences, Zahedan, Iran.; ^4^Department of Cell and Molecular Biology, Kosar University of Bojnord, Bojnord, Iran.; ^5^Department of Clinical Biochemistry, School of Medicine, Zahedan University of Medical Sciences, Zahedan, Iran.

**Keywords:** Endometriosis, Apoptosis, Genetic polymorphism, Bax, Bcl-2.

## Abstract

**Background:**

Endometriosis is a chronic, gynecological disorder, and the disease's pathogenesis is still debatable. Genes related to apoptosis have been revealed to be deregulated in endometriosis.

**Objective:**

This study investigates the relationship between polymorphic variants of *Bax -248G > A *and* Bcl-2 *-938C > A promoter regions with endometriosis risk in an Iranian population.

**Materials and Methods:**

In this case-control study, the polymorphisms of *Bax* -248G > A and *Bcl*-2 -938C > A promoter regions were analyzed in 127 Iranian cases and 125 controls who were referred to Ali-ibn-Abi Taleb Educational hospital, Zahedan, Iran between May 2022 and February 2023. The genotypic analysis was performed for all the subjects using the polymerase chain reaction-restriction fragment length polymorphism method.

**Results:**

The frequencies of mutant allele A carriers and the A allele of *Bax* -248G > A polymorphism showed about 2-fold significant increase of endometriosis risk (p = 0.04; p = 0.01, respectively). The frequencies of the mutant genotype AA and A allele carriers of *Bcl-2 *-938C > A polymorphism were approximately 4 and 2.5-fold higher in endometriosis compared to the control women, which were highly significant (p > 0.001). Moreover, the allele A frequency of *Bcl-2 *-938C > A was associated with a 2-fold higher risk of endometriosis (p > 0.001). Furthermore, the combination effects of these 2 single nucleotide polymorphisms showed that women with *Bax -248G > A* GGand *Bcl-2 -938C > A* AA variant alleles were associated with about 5 times higher risk of endometriosis (p > 0.001). Notably, a significant difference was observed in mutant allele distribution between minimal/mild (stage I and II) and moderate/severe (stage III and IV) women with endometriosis disease.

**Conclusion:**

The results of our study provide evidence that *Bcl-2* -938C > A and *Bax *-248G > A single nucleotide polymorphisms might be associated with the risk of endometriosis.

## 1. Introduction

Endometriosis is a chronic, benign, gynecological, heritable, condition that is considered by extrauterine growth of endometrial tissue, and it has been shown by mysterious etiology (1). The disease influences 6–10% of women of reproductive age (2) and results in severe and chronic pelvic pain, dyspareunia, dysmenorrhea infertility, and dyspareunia (3). The occurrence of endometriosis is 20–40% in infertile women, showing a harmful impact on the pregnancy rate of in vitro fertilization. Twin and family studies have revealed that genetic factors are related to endometriosis pathogenesis (4). Our knowledge about the etiology and pathophysiology of endometriosis is unclear yet. Nevertheless, the genetic aspects of endometriosis might assist in around half the variation in the susceptibility to the disease.

Genes related to apoptosis have been revealed to be deregulated in endometriosis. Electron microscopic analyses have determined the existence of apoptotic bodies in endometrial tissue through the late-secretory phase with a significant reduction of endometrial cell apoptosis in endometriosis.

It has been shown that spontaneous apoptosis had decreased in the ectopic endometrium in comparison to the eutopic endometrium of endometriosis individuals. Besides, the apoptosis level were reduced in the eutopic endometrium of endometriosis women compared with control group. Therefore, the decrease response of endometrial tissue to the apoptosis pathway might be related to the development of endometriosis (5).

The possible mechanism for the reduction of apoptosis is associated with the well-known regulators of apoptosis, anti-apoptotic B-cell lymphoma-2 (Bcl-2) protein, which is bound to pro-apoptotic Bcl-2 associated X protein (Bax). Bax is a death-promoting member of the Bcl-2 family and induces apoptosis via hetero-dimerization with Bcl-2 protein. The apoptosis rate is determined by the relative level of expression of these 2 proteins, which interact physically with each other (6). The *Bax *gene is located on chromosome 19q13.3 with a promoter with four p53 protein binding sites and 6 exons (7). The* Bcl-2* gene is another member of the Bcl-2 family, which unlike the Bax protein inhibits apoptosis. It is mapped on chromosome 18q21.3, with 2 promoters, 3 exons, and 2 introns. Bcl-2 protein is situated on the mitochondrial outer membrane and prevents Bax from changing the membrane permeability (8).

Previous studies have identified novel single-nucleotide polymorphisms (SNP) in *Bax* and *Bcl-2* genes which are crucial factors in cell-cycle control and cell survival. A promoter SNP in the *Bcl-2 *gene, the -938C > A genotype (*rs2279115*), is identified to be associated with high *Bcl-2* gene expression and is related to some cancer developments (9–12). Another SNP mapped on the 5 ' untranslated region of the *Bax* promoter, -248G > A (*rs4645878*), has been defined to be linked to both downregulation of Bax protein (12) and different susceptibility to numerous cancers (13–17). To our knowledge, there is no report examining the association of *Bax *-248G > A and* Bcl-2 *-938C > A SNPs with endometriosis. This work aimed to study the polymorphic variants of these proapoptotic and antiapoptotic genes, *Bax *-248G > A, *rs4645878 *and* Bcl-2 -*938C > A,* rs2279115 *SNPs, with endometriosis susceptibility in a sample of the Iranian population.

## 2. Materials and Methods

### Study population

In this case-control association study, from May 2022 to February 2023, 252 women were enrolled (127 cases and 125 controls) from the Department of Obstetrics and Gynecology of Ali-ibn-Abi Taleb Educational hospital, Zahedan, Iran. The control group was age and sex-matched healthy women of the same ethnic background with regular menstrual cycles and had not received any hormone therapy in the past 6 months. The medical records, including laparoscopy, laparotomy for diagnosis, therapy of endometriosis, and other complementary diagnostic tests were collected by the clinicians and pathologists. The diagnosis of endometriosis was histologically inveterate via the presence of an endometrial gland and/or stroma in the lesions. The characteristics of the endometriosis group and controls are shown in table I. The mean ages of the endometriosis and control groups were 30.14 ± 7.22 and 31.28 ± 6.87 yr, respectively. The participant in the healthy group had no previous medical record of endometriosis. Individuals were excluded if they withdrew consent, non-Iranian citizenship, and/or previous medical history of chronic pelvic pain, dysmenorrhea, or dyspareunia.

### Extraction of DNA and genotyping analysis

**Figure 1:**
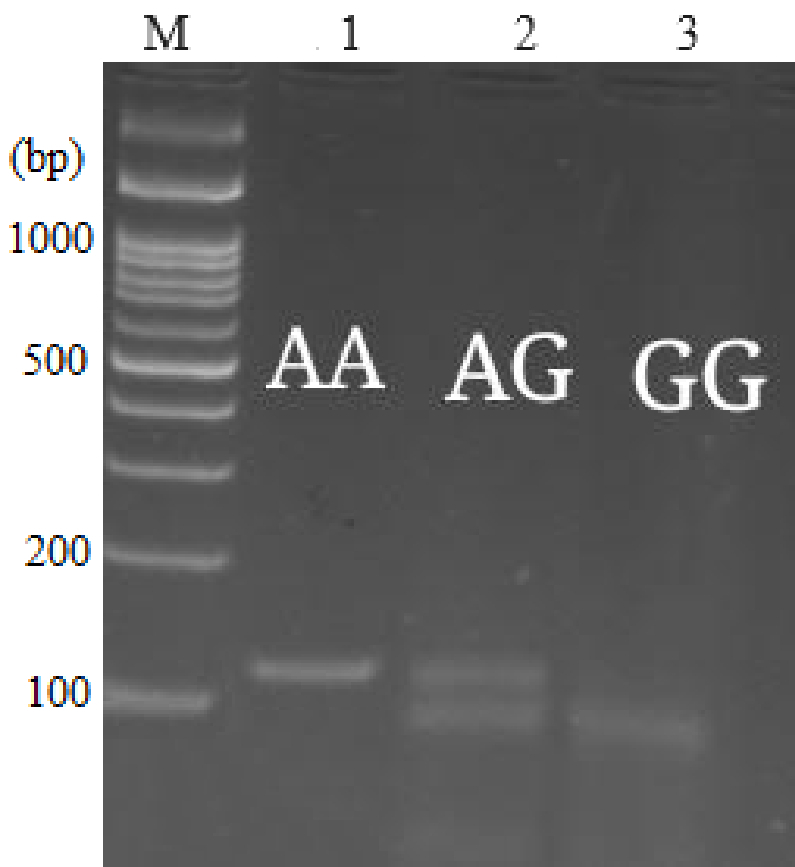
Genotyping of the *Bax* -248G > A rs4645878 polymorphism by PCR-RFLP. Lane M: 100 bp DNA ladder, Lane 1: Mutant homozygote AA (109 bp), Lane 2: Heterozygous AG (109 + 89 +20 bp), Lane 3: PCR wild homozygote GG (89 +20 bp).

**Figure 2:**
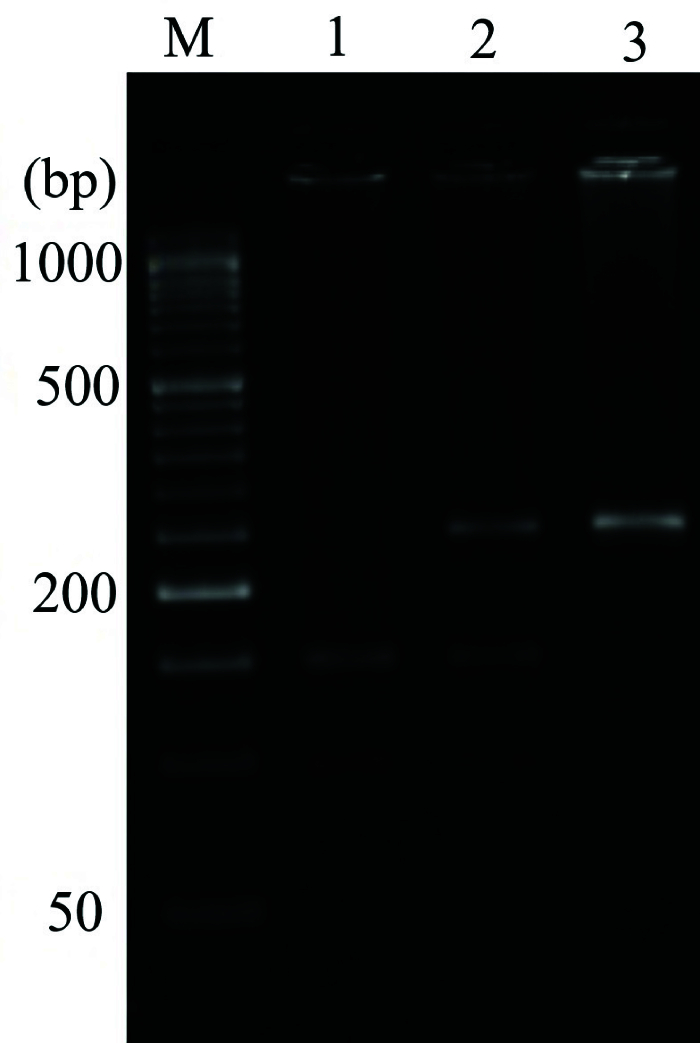
Genotyping of the *Bcl-2 *-938C > Ars2279115 polymorphism by PCR-RFLP. Lane M: 50 bp DNA ladder, Lane 1: Mutant homozygote AA (149 + 103 bp), Lane 2: Heterozygous CA (252 + 149 + 103 bp), Lane 3: PCR wild homozygote CC (252 bp).

The whole blood of the participants, case and controls, were collected in ethylenediaminetetraacetic acid-containing tubes. After DNA extraction using a commercial kit (DynaBio, Takapoozist, Iran) as followed by manufacturer's instructions, the all of extracted DNA was stored preoperatively in microtubes at -20 C until analyzed. The allelic discrimination of the *Bax* -248G > A and *Bcl*-2 -938C > A gene SNPs, *rs4645878 *and* rs2279115*, respectively, were analyzed by the polymerase chain reaction (PCR)-restriction fragment length polymorphism. The PCR primers of 2 SNPs were synthesized. The PCR program detail has been described in the previous study (18). The *MspI* and *BccI* restriction enzymes were utilized to digest the *Bax* and *Bcl*-2 PCR products, as followed by separation via 3% agarose gel electrophoresis. Each assay was run with negative controls. A random 20% of the samples were genotyped again to obtain accurate and reproducible results. The PCR products are shown in figures 1 and 2.

### Sample size

The sample size was calculated by online version of sample size calculator server (https://clincalc.com/stats/SampleSize.aspx), where P1 was 3%, representing the frequency of the mutant allele in control, P2 was 14%, which signifies the frequency of the mutant allele in case, with study power set to 80% and 95% of confidence intervals with the obtained critical value of < 2.0. P1 and P2 were determined based on the previously reported percentages (19). The minimum sample size was adjusted for a total of 200 subjects.

**Table 1:** The characteristics of endometriosis cases and controls


**Clinical characteristics**		**Case (n = 127)**	**Control (n = 125)**	P-value*
Age (Yr)*		30.14 ± 7.22	31.28 ± 6.87	0.20
BMI (kg/m^2^)*		22.57 ± 4.11	23.04 ± 3.75	0.34
Menstrual cycle length (day)*		28.64 ± 4.31	29.18 ± 3.86	0.29
Menstrual period length (day)*		5.79 ± 1.15	5.66 ± 0.96	0.33
Symptoms**				
	**Chronic pelvic pain**	65 (51.18)	17 (13.6)	< 0.001
	**Dysmenorrhea**	58 (45.66)	9 (7.2)	< 0.001
	**Dyspareunia**	49 (38.58)	21 (16.8)	< 0.001
Deliveries**				
	**Yes**	58 (45.66)	94 (75.2)	0.01
	**1**	19 (14.96)	51 (40.8)	< 0.001
	**2 and more**	39 (30.70)	43 (34.4)	0.65
	**No**	69 (54.33)	31 (24.8)	< 0.001
Spontaneous abortions**				
	**Yes (spontaneous)**	5 (3.93)	4 (3.2)	0.72
	**No**	122 (96.06)	121 (96.8)	0.96
Clinical stage**				
	**Minimal/mild (I and II)**	74 (58.26)		
	**Moderate/severe (stage III and IV)**	53 (41.73)		
*Data presented as Mean ± SD, Student *t* test. **Data presented as n (%), Student *t* test, and Chi-square test. BMI: Body mass index, Participants can have more than 1 symptom				

### Ethical considerations

The study protocol was approved by the Ethics Committee of the Zahedan University of Medical Sciences, Zahedan, Iran (Code: IR.ZAUMS.REC.1401.073), and performed according to the guidelines of the Declaration of Helsinki. Informed consent was obtained from all participants.

### Statistical analysis

Statistical analyses were performed using the Statistical Package for the Social Sciences, version 21.0 (SPSS Inc, Chicago, IL). Student's *t* test was used to detect differences in baseline clinical characteristics between cases and healthy women. Hardy-Weinberg equilibrium in both groups was examined with the Chi-square test to ensure they were representative. Differences in genetic distribution between cases and healthy women were compared using Pearson's Chi-square test. We calculated OR and 95% CI to reveal the relative risk degree. The p-values < 0.05 were regarded as significant.

## 3. Results

### Demographic and clinical characteristics

This study enrolled 125 infertile endometriosis women and 127 healthy controls without any history of endometriosis. The average ages of the case and the control group were not significantly different. A study of endometriosis-associated symptoms and the number of deliveries among the case and the control group revealed significant differences between them. Symptoms such as chronic pelvic pain, dysmenorrhea, and dyspareunia, were more frequent among endometriosis women (p < 0.001). No significant difference was found in body mass index, menstrual cycle length and period length, and spontaneous abortions among case and control groups. According to the data in the table, a significant difference was observed in the number of deliveries between the patients and the control group. Especially in the delivery of one child (p < 0.001). Based on the clinical stages, 58.26% of cases had minimal/mild (I and II) endometriosis and 41.73% of them had moderate/severe (stage III and IV) endometriosis (Table I).

### Frequency distributions of *Bax* -248 G > A genotypes and alleles among endometriosis and control groups

The genotypes and allele distributions of *Bcl-2* and *Bax* SNPs in endometriosis women and controls are listed in table II. The genotype frequencies of this SNP were in agreement with Hardy-Weinberg equilibrium in the case and healthy women (p = 0.03, and 0.38, respectively). No association in genotypes GG, GA, and AA was observed between case and control groups. Under the dominant model (GG vs. GA+AA), we observed a significant association between *Bax* -248G > A variant and about 2-fold higher risk of endometriosis in contrasted genetic models. Interestingly, investigation of allele frequencies at *Bax*-248G > A,* rs4645878, *revealed significant increases of the mutant allele A, approximately 2-fold, in cases compared with the controls (p = 0.01) (Table II).

### Frequency distributions of *Bcl*-2 -938C > A genotypes and alleles among endometriosis and control groups

Our data showed that the frequency of the *Bcl-2 * -938C > A, *rs2279115*, homozygous mutant variant AA was significantly higher in cases compared to controls (OR: 3.859; p = 0.001). In addition, under the dominant CA and AA combined vs. CC model, a significantly higher risk of endometriosis was observed. Furthermore, under the recessive model (AA vs. CC+CA), a significant difference of *Bcl-*2 -938C > A gene polymorphism with approximately 3-fold increase of endometriosis risk was observed. Interestingly, investigation of allele frequencies revealed significant increases, approximately 2-fold, of the mutant allele A in endometriosis women compared with the controls (Table II).

### Combination effects of *Bax* -248G > A and *Bcl*-2 -938C > A gene polymorphism on endometriosis risk

After that we combined these 2 SNPs for association with endometriosis. Our data showed that the *Bax* -248G > A *rs4645878 *GG and*, *the *Bcl*-2 -938C > A *rs2279115* AA were classified as adverse genotype combinations based on association with about 5 times higher risk of endometriosis. In addition, we found that the combination of the *rs4645878* GG with, the *rs2279115* CC or *rs2279115* CA, significantly increased the risk of endometriosis. No statistically significant association was observed between other genotypic combinations of 2 SNPs (Table III).

### Association between *Bax* -248G > A and *Bcl*-2 -938C > A gene polymorphisms with clinical stages in endometriosis women 

While women with minimal/mild endometriosis and moderate/severe endometriosis were considered distinctly, there were some differences for *Bax* -248G>A rs4645878 and, *Bcl*-2 -938C>A rs2279115 SNPs. Considering the *Bax* -248G > A SNP, there was a significant difference in A allele distribution between minimal/mild (stage I and II) and moderate/severe (stage III and IV) women which revealed that A allele was found about 2.5-fold frequent in moderate/severe endometriosis than minimal/mild one. On the other side, moderate/severe endometriosis risk was about 5 times greater in cases with *Bcl-2* -938 C > A CA+AA genotype, which proposed that A allele might be a high-risk allele and could have a special effect on the severity of endometriosis disease (Table IV).

**Table 2:** Genotype and allele frequencies of *Bax* -248 G > A and *Bcl-2* -938 C > A gene polymorphism in controls and endometriosis women


**Genotype**		**Case (n = 127)**	**Control (n = 125)**	**P-value**	**OR (95% CI)**
***Bax *** **-248G > A**					
	**GG**	91 (71.65)	103 (82.40)	Ref.	
	**GA**	29 (22.83)	20 (16)	0.12	1.64 (0.869–3.099)
	**AA**	7 (5.51)	2 (1.6)	0.09	3.96 (0.80–19.55)
**Dominant**					
	**GG**	91 (71.65)	103 (82.40)		
	**GA+AA**	36 (28.34)	22 (17.60)	0.04	1.85 (1.01–3.37)
**Recessive**					
	**GG+GA**	120 (94.48)	123 (98.40)		
	**AA**	7 (5.51)	2 (1.60)	0.11	3.58 (0.73–17.61)
**Alleles**					
	**G**	211 (83.07)	226 (90.4)		
	**A**	43 (16.92)	24 (9.6)	0.01	1.91 (1.12–3.27)
	**HWE**	0.03	0.38		
***Bcl*** **-2 -938C > A**					
	**CC**	49 (38.58)	74 (59.2)		
	**CA**	55 (43.30)	42 (33.6)	0.01	1.97 (1.15–3.39)
	**AA**	23 (18.11)	9 (7.2)	< 0.001	3.85 (1.64–9.03)
**Dominant**					
	**CC**	49 (38.58)	74 (59.2)		
	**CA+AA**	78 (61.41)	51 (40.8)	< 0.001	2.30 (1.39–3.82)
**Recessive**					
	**CC+CA**	104 (81.88)	116 (92.80)		
	**AA**	23 (18.11)	9 (7.20)	0.01	2.85 (1.26–6.43)
**Alleles**					
	**C**	153 (60.23)	190 (76)		
	**A**	101 (39.76)	60 (24)	0.001	2.090 (1.424–3.068)
	**HWE**	0.27	0.37		
Data presented as n (%). Pearson's Chi-square test. HWE: Hardy-Weinberg equilibrium					

**Table 3:** The combination effects of *Bax* -248 G > A and *Bcl-2* -938 C > A gene polymorphism on endometriosis risk


***Bax*** ** -248 G > A**	***Bcl*** **-2 -938C > A**	**Case (n = 127)**	**Control (n = 125)**	**P-value** * *	**OR (95% CI)**
**GG**	CC	30 (23.62)	62 (49.60)	Ref.	
**GG**	CA	45 (35.43)	34 (27.20)	< 0.001	2.73 (1.46–5.10)
**GG**	AA	16 (12.59)	7 (5.60)	< 0.001	4.72 (1.75–12.70)
**GA**	CC	17 (13.38)	11 (8.80)	< 0.001	3.19 (1.33–7.66)
**GA**	CA	7 (5.51)	7 (5.60)	0.20	2.06 (0.66–6.42)
**GA**	AA	5 (3.93)	2 (1.60)	0.05	5.16 (0.94–28.19)
**AA**	CC	2 (1.57)	1 (0.8)	0.25	4.13 (0.36–47.41)
**AA**	CA	3 (2.36)	1 (0.8)	0.12	6.20 (0.61–62.13)
**AA**	AA	2 (1.57)	0	0.13	10.24 (0.4–11.23)
Data presented as n (%). Pearson's Chi-square test					

**Table 4:** Association between *Bax* -248G > A and *Bcl-2 *-938C > A gene polymorphism with clinical stage frequency in endometriosis women


**SNPs (genotype)**		**Clinical stage**			
		**Minimal/mild (I and II)**	**Moderate/severe (stage III and IV)**	**P-value**	**OR (95% CI)**
***Bax*** ** -248G > A**					
	**GG**	59 (79.72)	32 (60.37)	Ref.	
	**GA+AA**	15 (20.27)	21 (39.62)	0.01	2.58 (1.17–5.68)
***Bcl*** **-2 -938C > A**					
	**CC**	38 (51.35)	11 (20.75)	Ref.	
	**CA+AA**	36 (48.64)	42 (79.24)	< 0.001	4.03 (1.80–9.01)
Data presented as n (%). Pearson's Chi-square test. SNPs: Single nucleotide polymorphisms					

## 4. Discussion

In the current study, we investigated the association of the SNPs in *Bax* and *Bcl-2* genes, involved in apoptosis, with the risk of endometriosis. The presented study is the first attempt to evaluate the role of *Bax* -248G/A and *Bcl-2 *-938C/A SNPs on the susceptibility to the disease. A statistically significant increased endometriosis rate was observed in cases with the *Bax* -248G > A variant (A allele) and GA+AA dominant genotype. Furthermore, cases with the *Bcl-2* -938C > A mutant A allele or AA variant and CA+AA vs. CC model exhibited an elevated risk of endometriosis. We also reported about 3 times or greater risk of endometriosis when a combination of *Bcl-2* -938C > A and *Bax* -248G > A were studied. There was a statistical association between *Bcl-2 *-938C/A and *Bax *-248G > A polymorphism and moderate/severe (stage III and IV) disease in these cases. It is worth noting that the percentage of cases studied is limited to stage III/IV endometriosis instead of stage I/II, which indicates that moderate to severe stages of the disease have a higher genetic burden compared to minimal or mild endometriosis.

The relationship between SNPs and the development of diseases has long been accepted. Recent improvements in molecular biology techniques have resulted in attention to SNPs and their impact on the risk assessment and development of diseases (20). Female fertility is strongly affected by genetic causes, and several reports have intended to recognize important genes in diseases that influence female fertility such as recurrent pregnancy loss, pre-eclampsia, fibroids, polycystic ovary syndrome, endometriosis, option rates (21), with a strong genetic basis (22). Apoptosis as a form of programmed cell death is very important to women's reproduction according to its involvement in the turnover of endometrial cells, remodeling of the uterine spiral arteries, embryonic development, and immunologic tolerance of maternal placental (23). The rate of apoptosis is reduced in the endometrial cells of women with endometriosis (24). Apoptosis is triggered by 2 different sources, intrinsic and extrinsic pathways, which ultimately lead to irreversible cell death (25).

*Bcl-2 *has 2 promoters with different functions in the regulation of its expression, that is, the P2 promoter is mapped on the translation start site and performs as a negative regulator of the P1 promoter (26). The common SNP in the *Bcl-2* gene was -938C > A (rs2279115) in P2 and its mutant allele displayed a significant increase in *Bcl-2 *promoter activity. High *Bcl-2* over-expression saves cells from programmed death via enhancement of the stability of the mitochondrial outer membrane; therefore, stopping the intrinsic apoptotic pathway (27). The different *Bcl-2* gene expression, in the eutopic endometrium of women with endometriosis, caused reduced apoptotic cells and subsequently resulted in abnormal endometrial cells survival in the ectopic site (28).

The expression of *Bax*, as a pro-apoptotic gene in the *Bcl-2* family, with a pro-apoptotic role in the mitochondrial pathway*, *has a significant impact on the release of apoptosis-related proteins. Although some reports revealed that *Bax* -248G > A was associated with the *Bax *protein expression (7), the exact effect of the SNPon protein expression is controversial. Related to the A allele, higher *Bax* mRNA and protein expression were reported to be associated with G alleles (29). Besides, another study revealed it caused lower transcriptional activity for *Bax *(30). As well as another study proposed that there was no association between the SNP and *Bax* transcriptional activity (31). Recent meta-analyses proposed that the downregulation of Bax protein is not connected to the poor prognosis for some cancers. Therefore, its polymorphism might not enhance cancer susceptibility and affect the prognosis of the tumors. In addition, *Bax* -248G > A and *Bcl-2* -938C > A SNPs have no direct association with overall cancer susceptibility nevertheless the relationship might have established ethnic specificity, and might be associated with increasing adverse prognosis of some cancers (32). Another study reported an increased susceptibility to leukemia having *Bcl-2* -938C > A and *Bax *-248G > A SNPs that influenced their response to treatment and overall survival. The data specified that there was an association between the mutant allele of *Bcl-2 -938C > A*C > A and the *Bax* -248G > A gene and a significantly increased risk of developing leukemia, compared to the wild-type allele (17). There are few studies on the relation of these polymorphisms to fertility-associated diseases. There are only 2 reports that study the effect of *Bcl-2 -938C > A* C > A and *Bax* -248G > A on women's fertility-related disorders.

In the first report, it was assessed if these SNPs are related to unexplained recurrent pregnancy loss risk in a Brazilian population (33). They propose that the *Bax -248G > A *AG genotype is a protective genotype for primary recurrent pregnancy loss and that the *Bcl*-*2 *-938 CC and C are risk factors for secondary recurrent pregnancy loss. Another study investigated the probable impact of the placental *Bax *and *Bcl-2* polymorphisms on pre-eclampsia susceptibility and found that there was no relationship between pre-eclampsia and placental *Bax rs4645878* and *Bcl-2 rs2279115* SNPs (18).

The main limitation of our report is the relatively small sample size of the endometriosis group, which could lead to reduction in the statistical power for identification of relations between the studied SNPs and the disease. Nevertheless, the limitation was the consequence of selection criteria because all endometriosis women who participated in this analysis were confirmed via laparoscopy and were classified based on the endometriosis stage with histologic confirmation. However, another study with larger sample sizes is needed to validate our results.

## 5. Conclusion

The results suggest that the *Bax *-248G > A, *rs4645878 *and* Bcl-2 *-938C > A,* rs2279115 *single nucleotide polymorphisms play a significant role in the pathogenesis of endometriosis in Iranian women. Our current study is just the first of this kind for proof-of-principle. Possibly, polymorphic variants of *Bax* -248G > A and *Bcl-2* -938C > A promoter regions might result in the altercations in transcription factor binding to the promoter, leading to the change of gene expressions, which might be associated with endometriosis.

##  Data availability

All related data and materials are available from the corresponding authors upon request.

##  Author contributions

DJ, FF, and MT conceived and designed the study. AEKh and MR did the data curation. SGhZ and DJ conducted the data analysis and drafted the initial manuscript. AEKh, FF, MR, and MT helped with the interpretation of the results and gave critical comments on the manuscript. All authors contributed to the final version of the manuscript.

##  Conflict of Interest

The authors declare that there is no conflict of interest.
